# Editorial: Editor’s feature: negative findings in pharmacogenetics and pharmacogenomics

**DOI:** 10.3389/fphar.2023.1267344

**Published:** 2023-08-08

**Authors:** José A. G. Agúndez, Pedro Ayuso, Luis A. Quiñones, Elena García-Martín

**Affiliations:** ^1^ Institute of Molecular Pathology Biomarkers, University of Extremadura, Cáceres, Spain; ^2^ Laboratory of Chemical Carcinogenesis and Pharmacogenetics, Department of Basic-Clinical Oncology (DOBC), Faculty of Medicine, University of Chile, Santiago, Chile; ^3^ Latin American Network for Implementation and Validation of Clinical Pharmacogenomic Guidelines (RELIVAFCYTED), Santiago, Chile

**Keywords:** pharmacogenomics (PGx), pharmacokinetics and drug metabolism (PDM), adverse drug effects, genome-wide association studies (GWAS), DNA sequencing, genotyping, negative findings

This Research Topic was proposed to gather relevant negative information that could balance publication bias and provide negative evidence that can be eventually used in the design of procedures, or the formulation of recommendations for pharmacogenetics or pharmacogenomics implementation. Publication bias constitutes a major problem that might lead to increasing costs and team efforts on studies that have already been carried out (although not published). Also, negative findings can increase the accuracy of many studies, simply by ruling out putative confounders, and are crucial in the development of clinical practice guidelines to discriminate which factors should be included in the prediction algorithms.

The Research Topic comprises six articles to which eighty-six authors have contributed. Throughout the Research Topic, GWAS studies, as well as case-control studies analyzing the putative effect of pharmacogenomics variation on the risk of developing adverse drug effects or spontaneous disorders are included. All these studies share common features such as a careful experimental design, the selection of SNPs or genes to be analyzed is based on solid scientific evidence and they are well-powered studies that provide conclusive negative or null-hypothesis evidence.

The GWAS study by Trompet et al. analyzed the putative influence of genetic variants on cardiovascular disease risk reduction in patients treated with statins. This group analyzed, in a first stage, two clinical trials plus six cohort studies comprising more than 10.700 individuals of European descent, which constitutes the largest GWAS of clinical cardiovascular response to statins to date. In the second stage, they analyzed the most promising 144 SNPs with *p*-values <5.0 × 10^−4^. Despite the large sample size and the comprehensive genetic study carried out, this study presents compelling evidence suggesting that genetic testing is unlikely to lead to significant improvements in the utilization of statins concerning coronary outcomes.

In a cohort composed of 212 patients with angioedema caused by angiotensin-converting enzyme inhibitors and angiotensin receptor blockers, recruited in Germany and Austria, Mathey et al. sequenced five genes, namely *SERPING1, F12, PLG, ANGPT1,* and *KNG1*, that were reported to carry pathogenic hereditary forms of angioedema. No gene variants causing hereditary angioedema were identified, and no association between angioedema caused by angiotensin-converting enzyme inhibitors and angiotensin receptor blockers and variants in the genes studied was identified. This study confirms preliminary findings ruling out a major effect of variability in the genes studied and angioedema caused by these drugs.

Another GWAS study was carried out by Attelind et al. in patients treated with apixaban. The study included 1,325 participants, aiming to identify genetic factors able to predict apixaban pharmacokinetics and to identify putative associations with the risk of developing bleeding and thromboembolic events. Also, a candidate gene study including the genes *ABCB1, ABCG2, CYP3A4, CYP3A5* and *SULT1A1* was carried out. No major association between genetic variants and the pharmacokinetics of apixaban was identified, although a marginal association with the missense SNP rs2231142 (Gln141Lys) in the *ABCG2* gene was identified. Regarding bleeding and thromboembolic events, no statistically significant associations were identified.

In a separate investigation conducted by Campos-Staffico et al. various single nucleotide polymorphisms (SNPs) within the genes *ABCB1, ABCG2, CYP2J2, CYP3A4*, and *CYP3A5* were analyzed. The study involved 2,364 patients receiving direct oral anticoagulants rivaroxaban or apixaban, and the risk of developing bleeding was assessed. The SNPs included variants with clinical and/or functional effects, with a high minor allele frequency in the studied population. No major associations were identified, although a minor effect of the *CYP3A5* rs776746 and the *ABCB1* rs4148732 SNPs was observed.

The study by Jimenez-Jimenez et al. analyzed the putative effect of genetic variability of the nitric oxide synthase gene (*eNOS* or *NOS3*) on the risk of developing idiopathic restless legs syndrome (RLS). The hypothesis is based on the observation that altered expression of *NOS1* was detected in the substantia nigra of RLS patients, as well as altered nitrite levels in RLS patients. Authors analyzed the frequencies for four *NOS3* gene variants in nearly six hundred individuals, including a promoter SNP related to increased expression, and two common missense SNPs. The main findings were that the frequencies of genotypes and allelic variants were not associated with the risk for RLS and were not influenced by gender, age, and positive family history of RLS.

Finally, McEvoy et al. carried out a systematic review, a meta-analysis, and a candidate gene study on the putative effect of CYP3A genetic variability and adverse effects, particularly peripheral neuropathy, caused by taxane chemotherapy. The systematic review indicated controversy on the putative effect of the CYP3A4*22 or the CYP3A5*3 variant alleles. However, neither the candidate gene study nor the meta-analyses revealed any major association with these variant alleles*.*

In sum, this Research Topic comprehensively addressed definitive negative findings that hold significant value in advancing the fields of Pharmacogenetics and Pharmacogenomics, ultimately contributing to their implementation into clinical practice.

